# MASTL is essential for anaphase entry of proliferating primordial germ cells and establishment of female germ cells in mice

**DOI:** 10.1038/celldisc.2016.52

**Published:** 2017-02-07

**Authors:** Sanjiv Risal, Jingjing Zhang, Deepak Adhikari, Xiaoman Liu, Jingchen Shao, Mengwen Hu, Kiran Busayavalasa, Zhaowei Tu, Zijiang Chen, Philipp Kaldis, Kui Liu

**Affiliations:** 1 Department of Chemistry and Molecular Biology, University of Gothenburg, Gothenburg, Sweden; 2 Monash Biomedicine Discovery Institute, Department of Anatomy and Developmental Biology, Monash University, Melbourne, Victoria, Australia; 3 The Key Laboratory of Reproductive Endocrinology (Shandong University), Ministry of Education, Jinan, China; 4 Institute of Molecular and Cell Biology (IMCB), A*STAR (Agency for Science, Technology and Research), Singapore, Republic of Singapore; 5 Department of Biochemistry, National University of Singapore (NUS), Singapore, Republic of Singapore

**Keywords:** anaphase, cell cycle, mitosis, MASTL, PP2A, primordial germ cells

## Abstract

In mammals, primordial germ cells (PGCs) are the embryonic cell population that serve as germ cell precursors in both females and males. During mouse embryonic development, the majority of PGCs are arrested at the G2 phase when they migrate into the hindgut at 7.75–8.75 dpc (days post coitum). It is after 9.5 dpc that the PGCs undergo proliferation with a doubling time of 12.6 h. The molecular mechanisms underlying PGC proliferation are however not well studied. In this work. Here we studied how MASTL (microtubule-associated serine/threonine kinase-like)/Greatwall kinase regulates the rapid proliferation of PGCs. We generated a mouse model where we specifically deleted *Mastl* in PGCs and found a significant loss of PGCs before the onset of meiosis in female PGCs. We further revealed that the deletion of *Mastl* in PGCs did not prevent mitotic entry, but led to a failure of the cells to proceed beyond metaphase-like stage, indicating that MASTL-mediated molecular events are indispensable for anaphase entry in PGCs. These mitotic defects further led to the death of *Mastl*-null PGCs by 12.5 dpc. Moreover, the defect in mitotic progression observed in the *Mastl*-null PGCs was rescued by simultaneous deletion of *Ppp2r1a* (α subunit of PP2A). Thus, our results demonstrate that MASTL, PP2A, and therefore regulated phosphatase activity have a fundamental role in establishing female germ cell population in gonads by controlling PGC proliferation during embryogenesis.

## Introduction

Primordial germ cells (PGCs) are embryonic cells that serve as progenitors of female and male gametes, and eventually differentiate into oocyte and sperm [1, 2]. In mice, the founders of PGCs originate from pluripotent proximal epiblast cells at 6.0–6.5 dpc (days post coitum) [3]. After receiving bone morphogenetic protein (BMP) signals from the extraembryonic ectoderm, these founder cells are identifiable as PGCs at 7.25 dpc and expression of *Dppa3* (*developmental pluripotency associated 3*; also known as *Pgc7* or *Stella*) marks the establishment of PGCs [4]. They begin to migrate through the developing hindgut and the mesentery, and eventually colonize the gonads at 10.5 dpc [5, 6]. In mice, PGCs in the migratory phase from 7.75 to 8.75 dpc are arrested at the G2 phase [7] and they proliferate rapidly from 9.5 to 12.5 dpc with a doubling time of 12.6 h [2, 8]. Mouse PGCs increase their numbers from 9.5 to 12.5 dpc by 50-fold because of this rapid proliferation [8]. By 13.5 dpc, PGCs in female mouse embryos undergo a mitotic–meiotic transition and give rise to female germ cells [9, 10]. In male mice, PGCs undergo mitotic arrest at G0/G1 after 13.5 dpc and meiosis starts only after birth, around post-natal day (PD) 10 [11, 12]. However, the molecular mechanisms regulating the cell cycle of proliferating PGCs between 9.5–12.5 dpc have not been investigated.

The mammalian cell cycle is driven by sequential activation of different types of cyclin-dependent kinases (CDKs) [13, 14], and genetic studies in mice have shown that Cdk1 is the most important Cdk for entry and progression through mitosis [15, 16]. However, an increase in the activity of Cdk1 alone is not sufficient for faithful mitotic progression [17] and a parallel suppression of phosphatase activity is equally crucial, as shown in *Xenopus* egg extracts and *Drosophila melanogaster* [18, 19]. Studies in human cell lines, mouse embryonic fibroblasts (MEFs), and *D. melanogaster* demonstrated that the activation of the Greatwall kinase (GWL) or its mammalian orthologue MASTL (microtubule-associated serine/threonine kinase-like) is essential for G2-M phase transition and mitotic progression [20–22]. In *Xenopus* egg extracts, it has been shown that activated GWL phosphorylates endosulfine α (ENSA) and cAMP-regulated phosphoprotein 19 (ARPP19), and converts them into potent inhibitors of PP2A (protein phosphatase 2A). Thus, phosphorylated ENSA/ARPP19 can bind to PP2A-B55 (PP2A with its regulatory subunit B55) and inhibit PP2A activity, which occurs at the same time when Cdk1 activity peaks [23–26]. These regulatory events ensure the maximal phosphorylation of Cdk1 substrates to complete mitosis as shown in *Xenopus* egg extracts [24].

In the current study, we investigated the functions of MASTL in PGC proliferation by using a tamoxifen-inducible *Dppa3*-*CreMER* (Cre fused with *murine estrogen receptor*) mouse model to delete *Mastl* from PGCs. We found that the deletion of *Mastl* from proliferating PGCs resulted in a significant loss of PGCs by 12.5 dpc. *Mastl*-null PGCs did not proceed to anaphase, indicating that MASTL is indispensable for anaphase entry in PGCs. This mitotic defect further led to the activation of the DNA damage response pathway and thus the majority of *Mastl*-null PGCs underwent apoptotic cell death by 12.5 dpc. Moreover, the anaphase entry defect in *Mastl*-null PGCs was rescued by the simultaneous deletion of *Ppp2r1a* (α subunit of PP2A). Thus, our results demonstrate that phosphatase regulatory unit MASTL-PP2A has a fundamental role in mediating mouse PGC proliferation.

## Results

### *Dppa3*-*CreMER* specifically deletes *Mastl* in PGCs

We used a tamoxifen-inducible *Dppa3*-*CreMER* mouse model to induce Cre activity specifically in PGCs [4]. We crossed *Dppa3-CreMER* mice with *mT/mG* reporter mice [27], and observed that in the embryo, Cre-expressing PGCs under the control of the *Dppa3* promoter exhibit a switch from red fluorescence (mT, membrane-targeted Tomato) to green fluorescence (mG, membrane-targeted green fluorescence protein, GFP). Injection of tamoxifen to pregnant females at 9.5 dpc caused the expression of mG specifically in female PGCs at 13.5 dpc. The specific Cre activity in PGCs was further confirmed by double immunofluorescence analysis of female embryonic gonads at 13.5 dpc using both anti-mouse Vasa homolog (MVH, a germ cell marker) and anti-GFP antibodies (Supplementary Figure S1C and F, arrows). We confirmed that the GFP-positive cells are indeed PGCs because these cells exclusively expressed both GFP (Supplementary Figure S1A and D, arrows) and MVH (Supplementary Figure S1B and E, arrows). However, GFP expression was absent in MVH-positive cells of vehicle-treated *Dppa3*-*CreMER*; *mT/mG* female embryonic gonads at 13.5 dpc (Supplementary Figure S1G–I, arrows).

We crossed *Mastl*^*loxP/loxP*^[28] female mice with *Mastl*^*loxP/loxP*^, *Dppa3-CreMER; mT/mG* male mice and tamoxifen was injected in pregnant females at 9.5 dpc (Supplementary Figure S1J). The resulting *Mastl*^*loxP/loxP*^, *Dppa3*-*CreMER; mT/mG* embryos were referred to as PGC-*Mastl*^−/−^. As a control, we crossed *mT/mG* female mice with *Dppa3*-*CreMER; mT/mG* male mice, and the resulting *Dppa3-CreMER; mT/mG* embryos were referred to as PGC-*Mastl*^+/+^.

**Figure 1:**
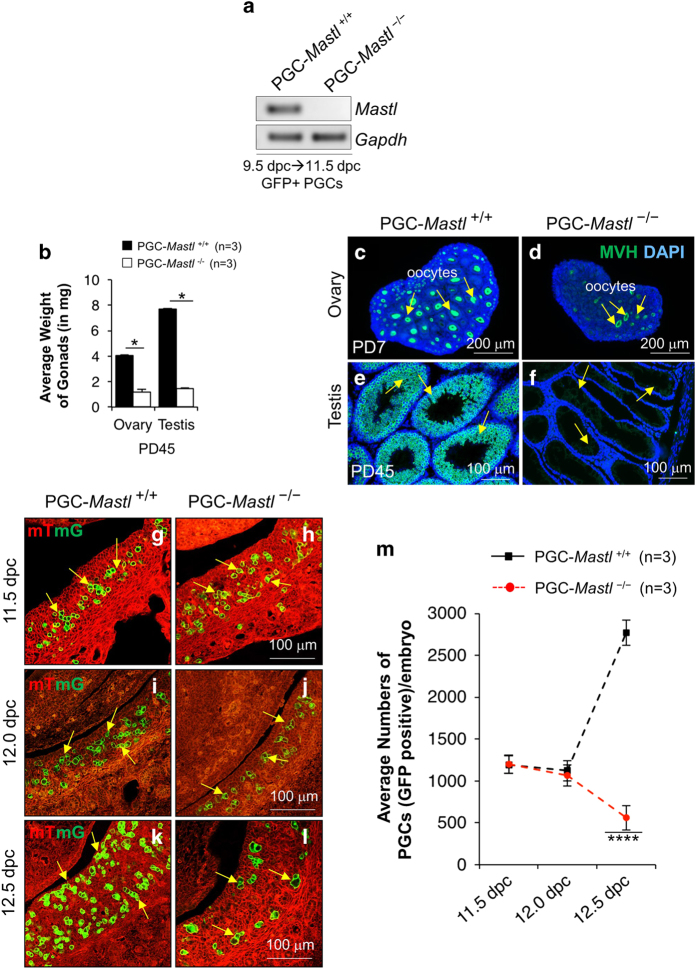
Deletion of *Mastl* in PGCs leads to the depletion of germ cells in both males and female gonads. (**a**) RT-PCR showing the absence of *Mastl* mRNA expression in 11.5 dpc *Mastl*^−/−^ PGCs, indicating successful deletion of *Mastl* gene in PGCs after tamoxifen injection at 9.5 dpc. *Gapdh* was used as an internal control. (**b**) Average weight (in mg) of adult PGC-*Mastl*^+/+^ and PGC-*Mastl*^−/−^ testes and ovaries at PD45. PGC-*Mastl*^−/−^ testes and ovaries were significantly smaller than PGC-*Mastl*^+/+^ testes and ovaries. ‘n’ is the number of female and male gonads analyzed for each genotype. (**c**, **d**) Staining for MVH (green) showing that by PD7 only a few follicles remained in the PGC-*Mastl*^−/−^ovary (**d**, arrows) compared to the PGC-*Mastl*^+/+^ ovary where a normal pool of follicles was seen (**c**, arrows). Scale bars=200 μm. (**e**, **f**) Staining for MVH (green) showing that PGC-*Mastl*^−/−^ seminiferous tubules in adult testes were deficient in germ cells (**f**, arrows) compared to PGC-*Mastl*^+/+^ testes (**e**, arrows) at PD45. Scale bars=100 μm. DNA was stained with DAPI. (**g**–**l**) Morphological analysis of female embryonic gonads showing that the deletion of *Mastl* in PGCs at 9.5 dpc caused the depletion of PGCs by 12.5 dpc. The morphology of 11.5 dpc (**g**, **h**, arrows), 12.0 dpc (**i**, **j**, arrows), and 12.5 dpc (**k**, **l**, arrows) GFP-positive *Mastl*^+/+^ and *Mastl*^−/−^ PGCs. Scale bar=100 μm. (**m**) Quantification of the average numbers of GFP-positive *Mastl*^+/+^ PGCs (black dotted line) and *Mastl*^−/−^ PGCs (red dotted line) per female embryo at 11.5, 12.0 and 12.5 dpc. At 11.5 and 12.0 dpc, no significant changes in the number of PGCs were seen in PGC-*Mastl*^+/+^ and PGC-*Mastl*^−/−^ female embryonic gonads. At 12.5 dpc, however, the PGC-*Mastl*^−/−^ female embryonic gonads displayed a significant reduction in PGCs compared to PGC-*Mastl*^+/+^ female embryonic gonads. ‘n’ is the number of female embryos analyzed for each genotype. The experiments were repeated three times each, for each time point gonads from one embryo of each genotype were used, and representative images are shown. The data are means±s.e.m., *P<0.05 and ****P<0.0001.

To validate the deletion of *Mastl* in 11.5 dpc female gonads, we used GFP to sort *Mastl*^+/+^ and *Mastl*^−/−^ PGCs by fluorescence-activated cell sorting (FACS) from 11.5 dpc female gonads. RT-PCR indicated that the *Mastl* mRNA expression was almost completely absent in *Mastl*^−/−^ PGCs (Figure 1a). These results indicated that *Dppa3-CreMER* led to efficient deletion of *Mastl* by tamoxifen injection at 9.5 dpc (Supplementary Figure S2).

### Ablation of *Mastl* in PGCs results in germ cell loss in the gonads

The PGC-*Mastl*^−/−^ female and male mice had significantly smaller ovaries and testes, respectively compared with PGC-*Mastl*^*+/+*^ mice at PD 45 (Figure 1b). The deletion of *Mastl* in PGCs resulted in a nearly complete loss of germ cells in both males and females in adulthood as shown by MVH staining for germ cells in ovaries and testes at PD7 and PD45, respectively (Figure 1c–f, arrows). In subsequent experiments, we focused our studies on the development of female PGCs.

We found that the average numbers of *Mastl*^−/−^ and *Mastl*^*+/+*^ PGCs were indistinguishable in 11.5 dpc (Figure 1g and h, arrows and m) and in 12.0 dpc female gonads (Figure 1i and j, arrows and m). However, analysis of 12.5 dpc female gonads revealed a significantly lower number of *Mastl*^−/−^ PGCs compared with *Mastl*^*+/+*^ PGCs (Figure 1k and l, arrows and m). These results indicated that by 12.5 dpc the majority of *Mastl*^−/−^ PGCs had been depleted.

### MASTL deficiency in PGCs causes deregulation of cell cycle

**Figure 2:**
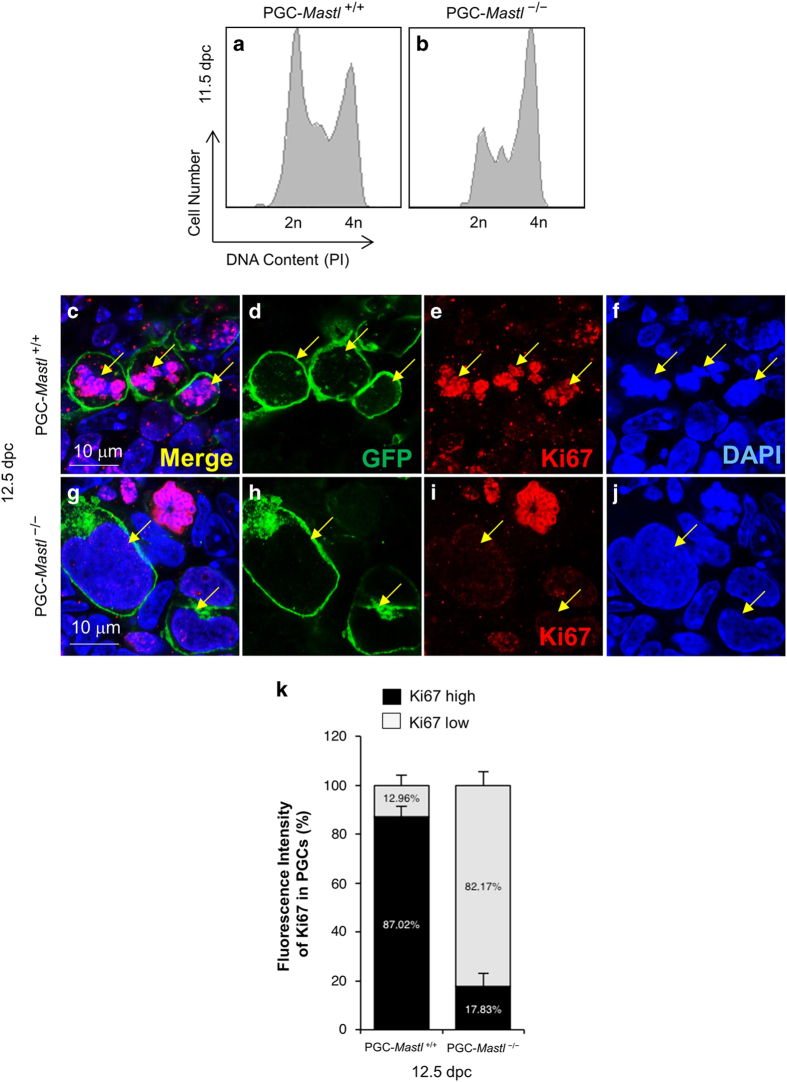
Deletion of *Mastl* in PGCs results in deregulation of the cell cycle. (**a**, **b**) FACS plots depicting the percentages of *Mastl*^+/+^ and *Mastl*^−/−^ PGCs with 2n (37.9 versus 30.3%) and 4n DNA content (39.4 versus 58.2%) at 11.5 dpc. The vertical axis represents the cell number and the horizontal axis represents the DNA content (PI staining). (**c**–**j**) Staining for the cell proliferation marker Ki67 (red) in *Mastl*^−/−^ PGCs (**g**, arrows) and *Mastl*^+/+^ PGCs (**c**, arrows). PGCs were co-stained with a GFP antibody (green) that recognizes mG (d, h, arrows). The DNA was stained with DAPI (blue) (**f**, **j**, arrows). Scale bars=10 μm. (**k**) Quantification of mean fluorescence intensity of Ki67 in *Mastl*^+/+^ and *Mastl*^−/−^ PGCs (in %). PGCs with high Ki67 fluorescence intensity are shown in black, and PGCs with low Ki67 fluorescence intensity are shown in grey. The Data are means±s.e.m. The experiments were repeated three times each, for each time point, gonads from one embryo of each genotype were used, and representative images are shown.

To understand the cell cycle distribution of *Mastl*^*+/+*^ and *Mastl*^−/−^ PGCs, we carried out propidium iodide (PI)-based FACS cell cycle analysis of PGCs in female gonads. Notably, our FACS data at 11.5 dpc showed an accumulation of 58% of the *Mastl*^−/−^ PGCs with a 4n DNA content (Figure 2a) compared with an accumulation of 39% of the *Mastl*^*+/+*^ PGCs with a 4n DNA content (Figure 2b). When we examined cell proliferation by staining for Ki67 at 12.5 dpc, *Mastl*^−/−^ PGCs displayed lower levels of Ki67 staining compared to *Mastl*^*+/+*^ PGCs (Figure 2c–j, arrows and k).

**Figure 3:**
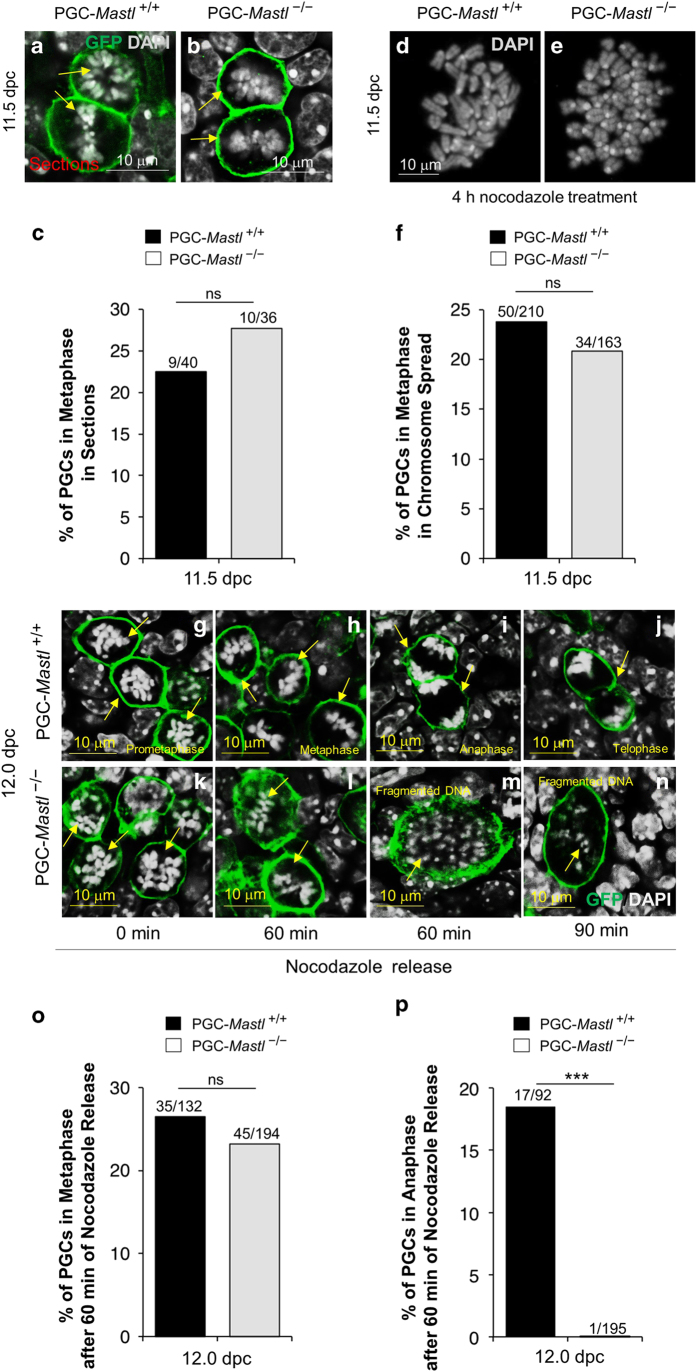
*Mastl*^−/−^ PGCs fail to transit to anaphase. (**a**, **b**) Morphological analysis of female embryonic gonads showing metaphase-like structures with condensed chromosomes stained with DAPI in GFP-positive *Mastl*^+/+^ and *Mastl*^−/−^ PGCs (arrows). (**c**) Quantification of the number of GFP-positive *Mastl*^+/+^ and *Mastl*^−/−^ PGCs displaying condensed chromosomes in sections. (**d**, **e**) Chromosome spreads of *Mastl*^+/+^ and *Mastl*^−/−^ PGCs after 4 h culture of 11.5 dpc female embryonic gonads in nocodazole showing metaphase-like chromosomes in *Mastl*^−/−^ and *Mastl*^+/+^ PGCs. Chromosomes were stained with DAPI. (**f**) Quantification of metaphase-like spreads per total number of cells at 11.5 dpc. The experiments were repeated three times each and representative images are shown. (**g**–**n**) Morphological analysis of female embryonic gonads demonstrating that both 12.0 dpc *Mastl*^+/+^ and *Mastl*^−/−^ PGCs were arrested in prometaphase after 4 h culture in nocodazole (**g**, **k**, arrows). 12.0 dpc *Mastl*^+/+^ and *Mastl*^−/−^ PGCs entered metaphase-like stage 60 min after nocodazole release (**h**, **l**, arrows). *Mastl*^−/−^ PGCs failed to enter anaphase (**m**, arrow) and displayed fragmented DNA structure, whereas *Mastl*^+/+^ PGCs progressed normally to anaphase (**I**, arrows) 60 min after nocodazole release. *Mastl*^−/−^ PGCs could not enter anaphase 90 min after nocodazole release (**n**, arrow), whereas *Mastl*^+/+^ PGCs had entered telophase (**j**, arrow). DNA was stained with DAPI. Scale bars=10 μm. (**o**, **p**) Quantification of 12.0 dpc *Mastl*^+/+^ and *Mastl*^−/−^ PGCs entering metaphase and anaphase 60 min after nocodazole release (in %). ‘n’ represents the total number of GFP-positive PGCs analyzed in three embryos of each genotype. The experiments were repeated three times each and representative images are shown. ***P<0.001.

Tissue sections of ovary (Figure 3a–c) and the metaphase spread of PGCs (Figure 3d–f) at 11.5 dpc displayed condensed chromosomes, indicating both *Mastl*^*+/+*^ and *Mastl*^−/−^ PGCs entered mitosis. We also found that more *Mastl*^−/−^ PGCs were positive for phosphorylated histone H3 at Ser10 (pHH3 S10, a mitotic marker) than *Mastl*^*+/+*^ PGCs (Supplementary Figure S3a–h, arrows and i). Thus, our results suggest that deletion of *Mastl* from PGCs at 9.5 dpc did not prevent mitotic entry, which differs from what was observed in *Xenopus* egg extracts.

### *Mastl* deletion prevents anaphase entry in PGCs

To study mitotic progression in *Mastl*^−/−^ PGCs, we took an *in vitro* approach and cultured 12.0 dpc female embryonic gonads in nocodazole-containing medium. Nocodazole is a compound that arrests the cells at prometaphase [29] due to its ability to destabilize microtubules, but upon release from nocodazole, cells synchronously enter metaphase and anaphase. We found that a 4-h treatment of 12.0 dpc female gonads with 0.2 μM nocodazole was sufficient to cause prometaphase arrest in wild-type PGCs (Supplementary Figure S4a, arrows and f). At 20 and 40 min after nocodazole release, the PGCs were still in prometaphase (Supplementary Figure S4b and c, arrows). However, at 60 min after release from nocodazole, 28.7% of the PGCs were in metaphase (Supplementary Figure S4d, arrows and g) and 13% were in anaphase (Supplementary Figure S4e, arrows and g).

We then arrested 12.0 dpc *Mastl*^*+/+*^ and *Mastl*^−/−^ PGCs in prometaphase for 4 h by nocodazole. Immediately after release from nocodazole arrest, both *Mastl*^*+/+*^ and *Mastl*^−/−^ PGCs were found in the prometaphase state (Figure 3g and k, arrows). When 12.0 dpc embryonic gonads were cultured with nocodazole followed by MG132 treatment to arrest PGCs at metaphase, we found that 47% of *Mastl*^−/−^ PGCs were arrested at metaphase-like stage (Supplementary Figure S4i and j), which was comparable to 48% of control *Mastl*^*+/+*^ PGCs at this stage (Supplementary Figure S4h and j). However, 60 min after release from nocodazole, 26.5% of the *Mastl*^*+/+*^ PGCs were in metaphase-like stage (Figure 3h, arrows and o) and 18.5% of the *Mastl*^*+/+*^ PGCs were in anaphase (Figure 3i, arrows and p). In contrast, 23.2% of the *Mastl*^−/−^ PGCs reached metaphase-like stage by 60 min after release from nocodazole (Figure 3l, arrows and o), but none of these mutant PGCs proceeded to anaphase (Figure 3p). The *Mastl*^−/−^ PGCs exhibited abnormal cellular morphology with fragmented DNA (Figure 3m, arrow). Even at 90 min after nocodazole release, the *Mastl*^−/−^ PGCs did not enter anaphase (Figure 3n), while the *Mastl*^*+/+*^ PGCs normally entered anaphase (Figure 3j), indicating that *Mastl*^−/−^ PGCs failed to enter anaphase and their DNA became fragmented, leading to a mitotic catastrophe.

We also observed that *Mastl*^−/−^ PGCs formed micronuclei (Supplementary Figure S5a and b, arrows and c), binucleate and giant cells (Supplementary Figure S5d, arrowheads), indicating chromosome segregation defects. Staining for phosphorylated ATM (pATM, Ser 1981) (Supplementary Figure S6a and b, arrows), Chk2 (Supplementary Figure S6c and d, arrows and e), and phosphorylated H2AX (γH2AX) (Supplementary Figure S6f and g, arrows and j) in 12.5 dpc *Mastl*^−/−^ PGCs indicated that DNA damage was increased in the absence of *Mastl* in PGCs. DNA damage caused a significant accumulation of p53 (Supplementary Figure S6h and i, arrows and k) and the downstream target of p53, PUMA (p53 upregulated modulator of apoptosis), in the nuleus of *Mastl*^−/−^ PGCs (Supplementary Figure S6l and m, arrows and p). As a response to the upregulation of p53 in *Mastl*^−/−^ PGCs, apoptotic pathways were activated since we observed increased staining for activated caspase-3 (Supplementary Figure S6n and o, arrows and q). Thus, the depletion of 12.5 dpc *Mastl*^−/−^ PGCs was associated with the activation of a caspase-3-dependent apoptotic pathway as a result of the metaphase arrest.

### The deletion of *PP2A* rescues the loss of *Mastl*^−/−^ PGCs

In *Xenopus* egg extracts, it has been shown that MASTL-mediated suppression of PP2A is essential for mitotic entry and progression [23, 24]. To investigate the contribution of PP2A activation in PGC development, we generated double-mutant mice lacking both *Ppp2r1a* (the PP2A *α*-subunit) and *Mastl* in PGCs (these embryos were referred to as PGC-*Mastl*^−/−^; *Ppp2r1a*^−/−^).

**Figure 4:**
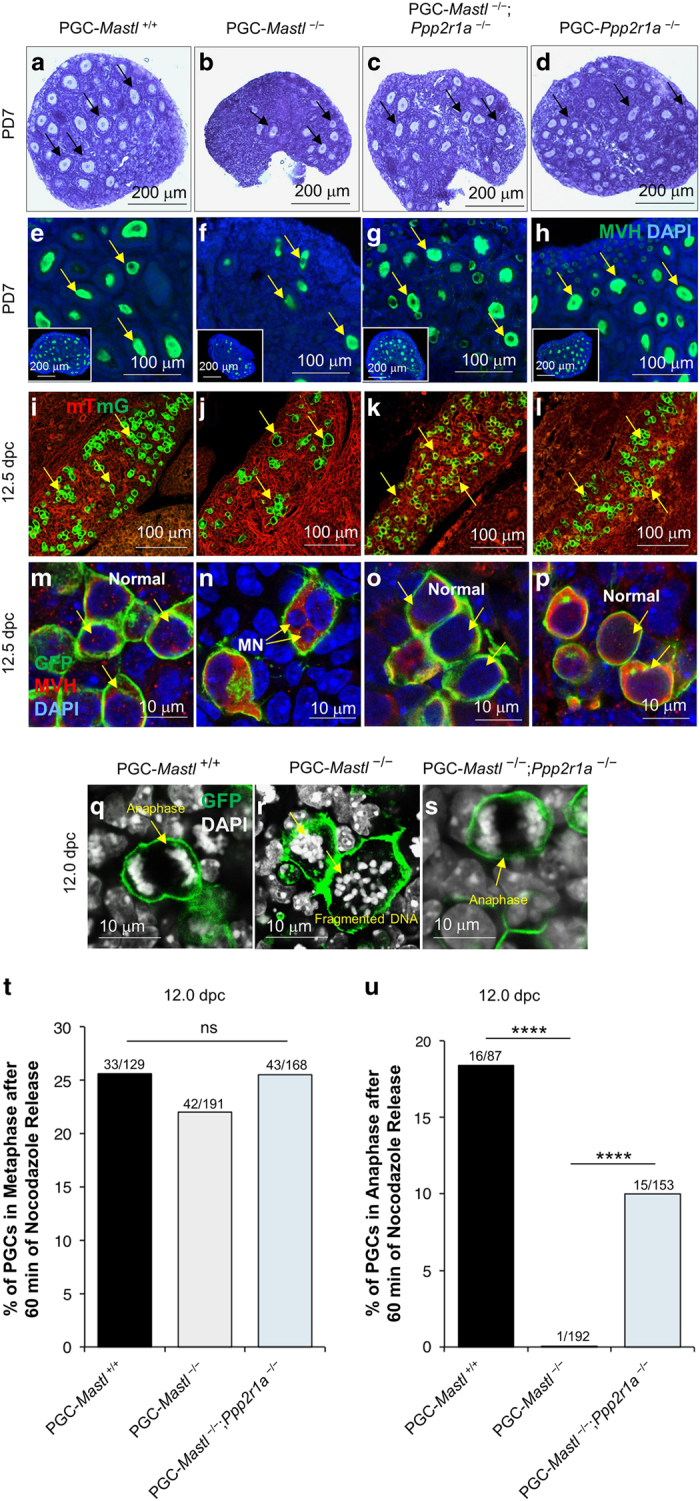
Deletion of *Ppp2r1a* in *Mastl*^*−/−*^ PGCs leads to anaphase entry. (**a**–**d**) Comparable ovarian morphologies in PGC-*Mastl*^+/+^ and PGC-*Mastl*^*−/−*^; *Ppp2r1a*^*−/−*^ female mice at PD7, indicating that the simultaneous deletion of *Mastl* and *Ppp2r1a* in PGCs (**a**, **c**, arrows) rescued the phenotype observed in PGC-*Mastl*^−/−^ ovaries (**b**, arrows). Scale bar=200 μm. (**e**–**h**) Staining for MVH (green) indicated that the simultaneous deletion of *Mastl* and *Ppp2r1a* in PGCs led to similar germ cell pools in PGC-*Mastl*^+/+^ and PGC-*Mastl*^*−/−*^; *Ppp2r1a*^*−/−*^ ovaries (**e**, **g**, arrows, a whole ovary is shown in the inset) compared with PGC-*Mastl*^−/−^ ovaries (**f**, arrows) at PD7. Scale bars=100 μm. (**i**–**l**) Morphological analysis of 12.5 dpc female embryonic gonads showed comparable numbers of PGCs in PGC-*Mastl*^+/+^ and PGC-*Mastl*^*−/−*^; *Ppp2r1a*^*−/−*^ female embryonic gonads (**i**, **k**, arrows) compared to fewer PGCs in PGC-*Mastl*^−/−^ gonads (**j**, arrows). Scale bars=100 μm. (**m**–**p**) Staining for MVH (red) and GFP (green) indicating that the simultaneous deletion of *Mastl* and *Ppp2r1a* in PGCs resulted in normal nuclear morphology in *Mastl*^*−/−*^; *Ppp2r1a*^*−/−*^ PGCs (**o**, arrows) similar to the nuclear morphology seen in *Mastl*^+/+^ PGCs (**m**, arrows). This is in sharp contrast to the nuclear morphology observed in *Mastl*^−/−^ PGCs (**n**, arrows; MN=micro nuclei). Scale bar=10 μm. (**q**–**s**) Morphological analysis of the simultaneous deletion of *Mastl* and *Ppp2r1a* in PGCs, which partially rescued the metaphase-anaphase transition defect after release from nocodazole arrest. After release from nocodazole, 12.0 dpc *Mastl*^*−/−*^; *Ppp2r1a*^*−/−*^ PGCs entered anaphase normally after 60 min, similar to *Mastl*^+/+^ PGCs (**q**, **s**, arrow). However, *Mastl*^−/−^ PGCs failed to enter anaphase and displayed fragmented DNA structures at 60 min after nocodazole release (**r**, arrows). (**t**, **u**) Quantification of 12.0 dpc *Mastl*^+/+^, *Mastl*^−/−^, and *Mastl*^*−/−*^; *Ppp2r1a*^*−/−*^ PGCs entering metaphase (**t**) and anaphase (**u**) after nocodazole release. DNA was stained with DAPI. Scale bars=10 μm. The experiments were repeated three times each, for each time point gonads from one embryo of each genotype were used, and representative images are shown. ****P<0.0001.

The simultaneous deletion of *Mastl* and *Ppp2r1a* appeared to rescue the ovarian phenotype observed in *Mastl*^−/−^ PGCs and resulted in normal-looking populations of follicles at PD7 in PGC-*Mastl*^*−/−*^; *Ppp2r1a*^*−/−*^ double knockout ovaries (Figure 4a–c and e–g, arrows, whole ovary in inset). We further confirmed that PGCs in *Mastl*^*−/−*^; *Ppp2r1a*^*−/−*^ female embryonic gonads developed and survived normally at 12.5 dpc (Figure 4i–k, arrows), and there were no abnormal nuclear structures or micronuclei (Figure 4m and o, arrows) such as those seen in *Mastl*^*−/−*^ PGCs at 12.5 dpc (Figure 4n). Additionally, the PGC-*Ppp2r1a*^*−/−*^ ovaries and PGC-*Ppp2r1a*^*−/−*^ embryonic gonads appreared normal, when *Ppp2r1a* was deleted from 9.5 dpc PGCs (Figure 4d,h,l and p, arrows).

When we cultured 12.0 dpc *Mastl*^*−/−*^; *Ppp2r1a*^*−/−*^ female embryonic gonads with 0.2 μM nocodazole for 4 h and released these gonads from prometaphase arrest for 60 min, 10% of the *Mastl*^*−/−*^; *Ppp2r1a*^*−/−*^ PGCs entered anaphase, compared to the *Mastl*^*−/−*^ PGCs (less than 1%) (Figure 4q–s, arrow and Figure 4t and u). Collectively, these results indicate that the loss of PP2A in *Mastl*^*−/−*^ PGCs partially rescued the mitotic exit defect.

### The absence of Cdk1 in PGCs causes mitosis arrest

Because phosphorylation of MASTL depends on Cdk1 activity in *Xenopus* egg extracts [30], we hypothesized that deletion of *Cdk1* from PGCs would phenocopy the *Mastl* deficiency in PGCs. We crossed *Cdk1*^*loxP/loxP*^ females [16] with *Cdk1*^*loxP/loxP*^; *Dppa3-CreMER; mT/mG* males and analyzed PGC development in the female embryonic gonads at 11.5 dpc, 12.5 dpc, and 13.5 dpc. We found that at 12.5 dpc, PGCs that were deficient in Cdk1 displayed significantly lower survival rate than *Cdk1*^*+/+*^ PGCs (Supplementary Figure S7a–d, arrowheads and g). By 13.5 dpc, *Cdk1*^*−/−*^ PGCs were almost completely absent from the gonads (Supplementary Figure S7e–f, arrowheads and g). Thus, our data indicate that *Cdk1*^*−/−*^ PGCs stop proliferating after 11.5 dpc, which results in severe depletion of PGCs by 13.5 dpc in PGC-*Cdk1*^*−/−*^ gonads. Moreover, lower levels of Ki67 staining indicated that *Cdk1*^*−/−*^ PGCs are not proliferating (Supplementary Figure S8a and b, arrowheads) and unable to progress through mitosis as indicated by the absence of pHH3 S10 immunostaining (Supplementary Figure S8c–e, arrowheads). These results were further supported by cell cycle analysis through FACS indicating that *Cdk1*^*−/−*^ PGCs were arrested at the G1 phase at 12.5 dpc (Supplementary Figure S8f and g). The failure of *Cdk1*^*−/−*^ PGCs to proceed into mitosis was accompanied with the upregulation of the apoptotic pathway at 12.5 dpc (Supplementary Figure S8h and i, arrowheads and j), which led to the elimination of defective PGCs. Together, our data show that the phenotype observed in *Cdk1*^*−/−*^ PGCs was not identical to that of *Mastl*^*−/−*^ PGCs, suggesting that Cdk1 has additional functions in PGCs besides activating MASTL.

## Discussion

**Figure 5:**
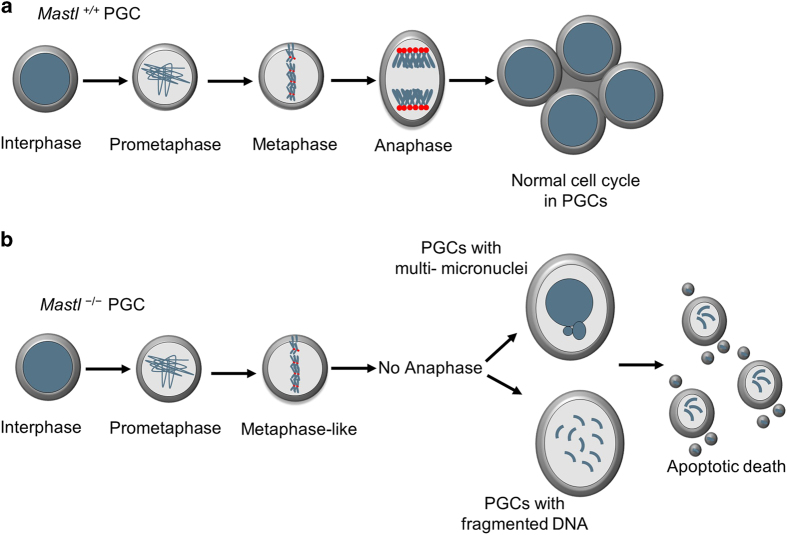
Model depicting cell cycle defect in *Mastl*-null PGCs. (**a**, **b**) In *Mastl*^+/+^ PGCs, the cell cycle progression is normal as shown in **a**. The loss of *Mastl* in PGCs causes defects in anaphase entry and abnormal *Mastl*^−/−^ PGCs are eliminated by apoptosis (**b**).

In the current study, we uncovered that MASTL has an essential role in PGC proliferation during embryogenesis, which is important for the establishment of oocyte pool in the mouse ovary. In the absence of *Mastl*, PGCs fail to exit mitosis, causing mitotic catastrophe and subsequent apoptotic cell death during embryonic development. Thus, *Mastl* is an indispensable gene for PGC development (as summarized in Figure 5a and b). Moreover, we found that simultaneous deletion of *Ppp2r1a* from PGCs partially rescued the defect in the mitotic exit caused by *Mastl* deletion, genetically demonstrating that MASTL-mediated suppression of PP2A activity is crucial for normal mitotic exit of proliferating PGCs.

MASTL has different roles during mitotic progression in different cell types, as shown by the diverse phenotypes reported in different model systems. In MEFs, loss of *Mastl* results in prometaphase arrest [20] and chromosome segregation defects [31]. In contrast, in *D. melanogaster, Gwl* or *Mastl* mutation generates chromosome condensation defects and a delay in mitotic entry and progression [22]. The RNAi knockdown of the *MASTL* gene in human cell lines causes G2 arrest and cytokinesis defects [21]. Hence, the *Mastl*^*−/−*^ PGCs provide a unique model to study how MASTL is involved in the dynamic control of the cell cycle in these specialized cell types, which are the precursors of female germ cells.

By using genetic approaches, we demonstrated that progression to prometaphase and metaphase in mouse PGCs is independent of MASTL and MASTL-mediated PP2A suppression. This is in sharp contrast to the phenotypes of *Mastl*-deficient MEFs, *D. melanogaster*, and human cell lines [20–22]. We further demonstrated that the failure to proceed beyond metaphase was likely caused by aberrantly elevated PP2A activity since simultaneous deletion of MASTL and PP2A led anaphase progression in PGCs. The failure in mitotic progression in *Mastl*^*−/−*^ PGCs led to the accumulation of DNA damage and the defective PGCs were quickly eliminated by apoptosis before the PGCs could differentiate into germ cells. Our results suggest that MASTL might safeguard DNA integrity during mitosis in rapidly dividing mouse PGCs.

PGCs are specified after receiving bone morphogenetic protein (BMP) signals from the extraembryonic tissues and they are among the first lineages to be established in embryos [32]. In addition, PGCs are unique embryonic cells that divide rapidly with doubling times as little as 12.6 h, thus expanding their population from ∼200 cells at 9.5 dpc to ∼10 000 cells at 12.5 dpc in mice [8]. Therefore, PGCs have a unique mitotic profile compared to most of the other mitotic cell types. However, there are only a limited number of studies on the mechanisms underlying the proliferation of PGCs. The loss of *Pin1* (*peptidyl-prolyl isomerase*) results in fewer germ cells in both male and female mice, and *Pin1*-null PGCs show proliferation defects due to a prolonged cell cycle and this leads to a reduced number of germ cells in the adult animal [33]. Additionally, *Pten* (*phosphatase and tensin homolog deleted from chromosome 10*) has been shown to be involved in cell cycle control of PGCs and loss of *Pten* causes the differentiation of PGCs into malignant cells and testicular teratoma in male mice [34]. Recently, it has been shown that *Prmt5* (*protein arginine methyltransferase 5*) is important for maintaining the cell cycle in PGCs in mice. The PGC-specific deletion of *Prmt5* causes cell cycle exit in PGCs and thus prevents the developmental switch that occurs between 9.5 and 10.5 dpc as marked by the expression of *Mvh* [35]. We believe that our findings are valuable for studying cell cycle regulation in PGCs in the context of the reported mechanisms of PGC proliferation.

In conclusion, our study demonstrated that *Mastl* is an indispensable gene involved in the mitotic progression in PGCs, which prevents mitotic catastrophe and the apoptotic cell death of PGCs. Mastl-mediated suppression of PP2A is essential to proceed into anaphase, as shown by the simultaneous deletion of *Mastl* and *Ppp2r1a* in PGCs compared with the deletion of *Mastl* alone. Therefore, this study expands our understanding of how the cell cycle is regulated during the rapid proliferation of PGCs in developing embryonic gonad.

## Materials and Methods

### Mice

*mT/mG* (007576) and *PPP2r1a*^*loxP/loxP*^ (017475) mice were purchased from the Jackson Laboratory (Bar Harbor, ME, USA). The *Dppa3-CreMER* (RBRC05385) mice were kindly provided by Mitinori Saitou of the Institute for Integrated Cell-Material Sciences, Kyoto University. The generation of the *Mastl*^*loxP/loxP*^ [28] and *Cdk1*^*loxP/loxP*^ [16] mice has been described previously. These *Mastl*^*loxP/loxP*^, *PPP2r1a*^*loxP/loxP*^, and *Cdk1*^*loxP/loxP*^ mice were crossed with *Dppa3-CreMER, mT/mG* mice to introduce the inducible Cre and *mT/mG* reporter system into the mice. After multiple rounds of crossing, we obtained homozygous *Mastl*^*loxP/loxP*^; *Dppa3-CreMER; mT/mG* mice (designated as PGC-*Mastl*^*−/−*^ mice), *Mastl*^*loxP/loxP*^; *PPP2r1a*^*loxP/loxP*^; *Dppa3-CreMER; mT/mG* mice (designated as PGC-*Mastl*^*−/−*^; *PPP2r1a*^*−/−*^ mice), *Cdk1*^*loxP/loxP*^; *Dppa3-CreMER; mT/mG* mice (designated as PGC-*Cdk1*^*−/−*^ mice), and *Dppa3-CreMER; mT/mG* mice (designated as PGC-*Mastl*^*+/+*^ mice and PGC-*Cdk1*^*+/+*^ mice). The obtained experimental mice were of mixed background (129S1/SvlmJ and C57BL/6).

All mice were housed under controlled environmental conditions with free access to water and food. Illumination was on between 6 a.m. and 6 p.m. Experimental protocols were approved by the regional ethical committee of the University of Gothenburg, Sweden.

Female *Mastl*^*loxP/loxP*^ mice were mated with male *Mastl*^*loxP/loxP*^; *Dppa3-CreMER; mT/mG* mice, and at noon on the day when a vaginal plug was observed was scored as 0.5 dpc. To delete *Mastl* from developing PGCs, we administered 2 mg tamoxifen (Sigma-Aldrich, St Louis, MO, USA, T5648) intraperitoneally to pregnant female mice at 9.5 dpc. The injected pregnant female mice were killed to harvest PGC-*Mastl*^*−/−*^ female embryonic gonads at 11.5 dpc, 12.0 dpc, and 12.5 dpc. A similar strategy was followed to generate PGC-*Mastl*^*+/+*^, PGC-*Mastl*^*−/−*^; *PPP2r1a*^*−/−*^, PGC-*Cdk1*^*+/+*^, and PGC-*Cdk1*^*−/−*^ embryos.

Embryos younger than 13.5 dpc were sexed by PCR for the X chromosome-specific gene *Jarid1c* and the Y chromosome-specific gene *Jarid1d* with the primers 5′- CTG AAG CTT TTG GCT TTG AG-3′ and 5′- CCA CTG CCA AAT TCT TTG G-3′, respectively. The PCR products were resolved in 3% agarose gels, and the male embryos showed a 331 bp band for *Jarid1c* and a 302 bp band for *Jarid1d,* whereas female embryos showed a single 331 bp band for *Jarid1c*.

### Preparation of tamoxifen

Tamoxifen (Sigma, T5648) was dissolved in 100% ethanol to make a stock solution of 100 mg ml^−1^ that was then diluted in corn oil (Sigma, C8267) prior to injection to make it 20 mg ml^−1^. The plugged females were injected with a single dose of 2 mg tamoxifen to induce excision of the floxed *Mastl* allele. The scheme for the injection of tamoxifen was optimized for the maximum survival of embryos and efficiency of *Mastl* deletion. A similar strategy was followed to induce excision of the floxed *PPP2r1a* and the floxed *Cdk1* alleles.

### Preparation of PGCs for FACS

Female *Mastl*^*loxP/loxP*^ mice were crossed with *Mastl*^*loxP/loxP*^*; Dppa3-CreMER; mT/mG* males to obtain *Mastl*^*loxP/loxP*^*; Dppa3-CreMER; mT/mG* embryos. Tamoxifen was injected into 9.5 dpc pregnant *Mastl*^*loxP/loxP*^ female mice, and PGC-*Mastl*^*+/+*^ and PGC-*Mastl*^−/−^ gonads were harvested from 11.5 and 12.5 dpc female embryos. The genotyping of *Mastl*^*loxP/loxP*^; *Dppa3-CreMER; mT/mG* embryos was done by visualizing whole gonads under a microscope (SteREO, Discovery. V8, Zeiss, Munich, Germany) for green fluorescence. The expression of GFP marked the Cre recombination and deletion of the *Mastl* gene in PGCs. Gonads were cleaned of non-gonadal tissues and digested for 30 min at 37 °C with 1 : 10 collagenase (400 U ml^−1^ collagenase IV stock (Gibco, Carlsbad, CA, USA) that was diluted 1 : 10 in 1× PBS). After 30 min of incubation, the collagenase was aspirated carefully without disturbing the gonads. The gonadal cells were dispersed in pre-warmed sterile 1× PBS by pipetting up and down several times. The GFP-positive *Mastl*^*+/+*^ and *Mastl*^−/−^ PGCs were sorted on a BD FACS AriaII cytometer (BD Biosciences, San Jose, CA, USA). The isolated/sorted GFP-positive PGCs were checked under a microscope, which showed a 99% pure population of PGCs. A similar strategy was followed for PGC-*Cdk1*^*−/−*^ and PGC-*Cdk1*^*+/+*^ embryos.

### RT-PCR

A total of 3000 *Mastl*^*+/+*^ and *Mastl*^−/−^ PGCs sorted by FACS were used to prepare total RNA with the RNeasy Mini kit (Qiagen, Hilden, Germany) according to the manufacturer’s instructions. Oligo (dT)-primed cDNA was synthesized using the iScript cDNA synthesis kit (BioRad, Hercules, CA, USA). Real time PCR for *Mastl* was performed using the primers 5′- GGA GTA TCT TAT TGG TGG AGA-3′ and 5′- AGC ATA TTG TCC GGT TTC AA-3′ on a CFX-Connect Real-Time system (Bio-Rad) using iTaq Universal SYBR Green PCR master mix (Bio-Rad). The reaction was performed in triplicate. *Gapdh* was used as the internal control. The PCR products were run on a 3% agarose gel with ethidium bromide.

### Cell cycle analysis

To determine the cell cycle distribution of *Mastl*^*+/+*^ and *Mastl*^−/−^ PGCs, *Mastl*^*loxP/loxP*^ females were crossed with *Mastl*^*loxP/loxP*^*; Dppa3-CreMER; mT/mG* males to obtain *Mastl*^*loxP/loxP*^*; Dppa3-CreMER; mT/mG* embryos. Tamoxifen was injected into 9.5 dpc pregnant *Mastl*^*loxP/loxP*^ female mice, and PGCs were isolated from 11.5 dpc PGC-*Mastl*^*+/+*^ and PGC-*Mastl*^−/−^ embryos. Briefly, the embryonic gonads were dispersed into single cells by incubating with 1 : 10 collagenase for 30 min at 37 °C. The individual GFP-positive *Mastl*^*+/+*^ and *Mastl*^−/−^ PGCs were sorted on a BD FACS AriaII cytometer (BD Biosciences). A total of 500–1000 PGCs were used for PI staining for 2 h using Vindelov’s reagent [36]. This reagent contained 100 ml Tris-buffered saline pH 7.6, 1 mg (350 units) ribonuclease A (Sigma, R-5000), 7.5 mg PI (Sigma, P4170), and 100 μl NP-40 (Sigma, N-3516). After PI staining, the BD FACS AriaII cytometer with FACSDiva software (BD Biosciences) was used for cell cycle analysis. A similar strategy was followed for 12.5 dpc PGC-*Cdk1*^*−/−*^ and PGC-*Cdk1*^*+/+*^ embryos.

### Histological analysis and immunofluorescence staining

*Mastl*^*loxP/loxP*^ females were crossed with *Mastl*^*loxP/loxP*^*; Dppa3-CreMER; mT/mG* males to obtain *Mastl*^*loxP/loxP*^*; Dppa3-CreMER; mT/mG* embryos. Tamoxifen was injected into 9.5 dpc pregnant *Mastl*^*loxP/loxP*^ female mice. Because tamoxifen injection compromised the natural vaginal delivery, *Mastl*^*loxP/loxP*^*; Dppa3-CreMER; mT/mG* pups were delivered by Cesarean section at 19.5 dpc. Ovaries and testes were dissected from adult PGC-*Mastl*^*+/+*^ and PGC-*Mastl*^−/−^ females and males (fostered mice), and were fixed in 4% paraformaldehyde (Sigma) at 4 °C overnight, embedded in paraffin, sectioned, and immunostained for rabbit MVH (Abcam, Cambridge, UK, ab13840) and mouse GFP (Santa Cruz Biotechnology, Santa Cruz, CA, USA, B-2, sc-9996). A similar strategy was followed to generate PGC-*Mastl*^*+/+*^, PGC-*Mastl*^*−/−*^; *PPP2r1a*^*−/−*^, PGC-*Cdk1*^*+/+*^ and PGC-*Cdk1*^*−/−*^ mice.

Immunofluorescence staining of embryonic sections was carried out as described previously [37]. Briefly, whole female embryos at 11.5 and 12.5 dpc were fixed at 4 °C overnight in 4% paraformaldehyde, dehydrated, paraffin embedded, and sectioned (8 μm thick). The slides were dewaxed and rehydrated with an ethanol gradient, and antigens were retrieved by microwaving in citrate buffer (10 mM sodium citrate, 0.05% Tween 20, pH 6.0) for 10 min. After blocking in 10% goat serum, slides were incubated with primary antibodies in 10% goat serum at 4 °C overnight. Slides were then incubated with goat secondary antibodies conjugated to Alexa 488 or Alexa 594 (Molecular Probes, Carlsbad, CA, USA), and DNA was stained with DAPI. The slides were viewed by using a Plan-Apochromat 63 X/1.4 Oil DIC M27 objective on a laser-scanning confocal microscope (Zeiss LSM 700).

Primary antibodies against rabbit phospho-histone H3 (S10) (Cell Signal Technology, Beverly, MA, USA, 9701), rabbit Ki67 (Abcam, ab15580), mouse p-ATM (S1981) (Santa Cruz Biotechnology, 10H11.E12, sc47739), rabbit Chk2 (Abcam, ab47433), rabbit γ-H2AX (S139) (Abcam, ab2893), mouse p53 (Abcam PAb 240, ab26), rabbit PUMA (Millipore, Billerica, MA, USA, ABC158), rabbit MVH/DDX4 (Abcam, ab13840), rabbit active cleaved caspase-3 (Abcam, ab2302), mouse GFP (Santa Cruz Biotechnology, B-2, sc-9996), and rabbit GFP (Abcam, ab290) were used in this study.

For Ki67 immunofluorescence staining, images were acquired with a Zeiss LSM 700 confocal microscope. The quantification of mean fluorescence intensity in GFP-positive PGCs was performed in Image J [38].

### Chromosome spread

Female embryonic gonads with mesonephroi were dissected from 11.5 dpc PGC-*Mastl*^*+/+*^ and PGC-*Mastl*^−/−^ embryos. The dissected embryonic gonads were cultured for 4 h in DMEM-F12 medium (Invitrogen, Carlsbad, CA, USA) supplemented with 15% FBS penicillin/streptomycin (Invitrogen), non-essential amino acids (Invitrogen), L-glutamine (Invitrogen), and sodium pyruvate (Invitrogen). The medium also contained 0.2 μM nocodazole (Sigma) to arrest PGCs in prometaphase. After culturing for 4 h, gonads were cleaned from non-gonadal tissues with a needle and incubated in 1:10 collagenase for 15 min at 37 °C. The collagenase-treated gonads were then incubated in 0.5% KCl for 10 min and disperesed in the remaining hypotonic buffer. Freshly prepared ice-cold acetic acid: methanol (1 : 3) fixative was used to fix the individualized PGCs. This step was repeated one more time and the cells were dropped onto slides. DAPI was used to analyze metaphase chromosome spread on a laser-scanning confocal microscope (Zeiss LSM 700).

### *In vitro* culture of embryonic gonads

For *in vitro* culture of embryonic gonads, *mT/mG* females were crossed with *Dppa3-CreMER; mT/mG* males to obtain *Dppa3-CreMER; mT/mG* transgenic embryos (wild type). All the experiments were carried out at room temperature unless otherwise indicated. Female embryonic gonads with mesonephroi were dissected from 12.0 dpc *Dppa3-CreMER; mT/mG* embryos (wild type). The dissected embryonic gonads were cultured for 4 h in DMEM-F12 medium (Invitrogen) supplemented with 15% FBS penicillin/streptomycin (Invitrogen), non-essential amino acids (Invitrogen), L-glutamine (Invitrogen), and sodium pyruvate (Invitrogen) along with 0.2 μM nocodazole to arrest PGCs in prometaphase. After 4 h of culturing, the female gonads were washed three times with pre-warmed culture medium and further cultured without nocodazole. At 0, 20, 40, and 60 min after release from nocodazole, 12.0 dpc *Dppa3-CreMER, mT/mG* female gonads were fixed in 4% paraformaldehyde overnight. These fixed 12.0 dpc female gonads were dehydrated, paraffin embedded, sectioned, and stained with DAPI to visualize the DNA. In a similar way, female gonads from 12.0 dpc PGC-*Mastl*^*+/+*^ and PGC-*Mastl*^−/−^ embryos were cultured in 0.2 μM nocodazole for 4 h. At 60 min after release from nocodazole, female gonads were fixed in 4% paraformaldehyde and processed as above. In these 12.0 dpc female gonads, PGCs were already expressing the mG reporter (GFP). For each experiment, three or more embryonic gonads were used for culture and analysis. A similar strategy was followed for generating PGC-*Mastl*^*−/−*^; *PPP2r1a*^*−/−*^ embryonic gonads.

To validate metaphase entry in *Mastl*^−/−^ PGCs, 12.0 dpc embryonic gonads from PGC-*Mastl*^*+/+*^ and PGC-*Mastl*^−/−^ embryos were cultured in 0.2 μM nocodazole for 4 h. After nocodazole washout, then embryonic gonads were cultured in 10 μM MG132 (Millipore) for 60 min. Then female gonads were fixed in 4% paraformaldehyde and processed as above.

### Statistical analysis

All experiments were repeated at least three times. The quantitative data (mean±s.e.m.) were analyzed by Student’s *t*-test by using GraphPad Prism version 6 (GraphPad Software, La Jolla, CA, USA), and differences were considered significant when *P*<0.05.
