# Supplementary material for: MASTL is essential for anaphase entry of proliferating primordial germ cells and establishment of female germ cells in mice
Source: Cell Discov. 2017 Feb 7;3:16052–. doi: 10.1038/celldisc.2016.52 (PMC5301161; doi:10.1038/celldisc.2016.52)
Supplement: Supplementary Information [file celldisc201652-s1.pdf]

Supplementary information

Figure S1. Inducible *Dppa3-CreMER* specifically deletes *Mastl* in mouse PGCs. (A–F) Validation of the specificity and efficiency of targeting PGCs with the *Dppa3-CreMER; mT/mG* mouse model. A single dose of tamoxifen was injected intraperitoneally into pregnant female mice at 9.5 dpc, and the embryonic gonads were analysed at 13.5 dpc. GFP (A and D, green, arrows) and MVH (B and E, red, arrows) indicated the specific recombination in PGCs. Scale bar = 200 µm. (**D–F**) Corresponding higher magnification images showing 13.5 dpc PGCs expressing both GFP (green, arrows) and MVH (red, arrows). Scale bars = 50 µm. **(G–I)** Vehicle-treated 13.5 dpc *Dppa3-CreMER; mT/mG* embryonic gonads stained with GFP (G, green) and MVH (H, red, arrows) antibodies, displayed no recombination in PGCs (I, arrows). Scale bar = 50 µm. **(J)** Schematic representation of the deletion of *Mastl* exon 4 by *Dppa3-CreMER* after tamoxifen injection at 9.5 dpc in PGCs. The experiments were repeated three times each, for each time point gonads from one embryo of each genotype were used, and representative images are shown.

Figure S2. PGC-specific recombination rate of *Dppa3-CreMER; mT/mG*. Recombination rate of *Dppa3-CreMER; mT/mG* in PGCs after tamoxifen and vehicle injection at 9.5 dpc. 'n' is the number of GFP-positive and MVH-positive PGCs analyzed after tamoxifen and vehicle injection in *Dppa3-CreMER; mT/mG* embryonic female gonads at 13.5 dpc.

Figure S3. Mitotic entry in *Mastl*$^{-/-}$ PGCs. (A–H) Staining for the mitotic marker pHH3 S10 (C and G, red, arrows) in 12.5 dpc *Mastl*$^{+/+}$ and *Mastl*$^{-/-}$ PGCs indicating that the deletion

of *Mastl* in PGCs did not affect mitotic entry. PGCs were co-stained with a GFP antibody that recognizes mG (B and F, green, arrows). The merged images of *Mastl*$^{+/+}$ and *Mastl*$^{-/-}$ PGCs (A and E, arrows). The DNA was stained with DAPI (D and H, blue, arrows). Scale bars = 10 µm. **(I)** Quantification of the number of GFP-positive *Mastl*$^{+/+}$ and *Mastl*$^{-/-}$ PGCs that were labeled for pHH3 S10 (in %). 'n' represents the total number of GFP-positive PGCs analyzed in three embryos of each genotype. The experiments were repeated three times each, and representative images are shown. Data are means ± SEM, ** $p < 0.01$.

Figure S4. The kinetics of cell cycle progression in wild-type PGCs and metaphase arrest by MG132 in *Mastl*-null PGCs. (A–E) Morphological analysis of GFP-positive PGCs (wild-type) indicating that culturing 12.0 dpc female gonads in 0.2 µM nocodazole for 4 hours resulted in prometaphase arrest (A, arrows). At 20 and 40 minutes after release from nocodazole arrest, PGCs were still in prometaphase (B and C, arrows) but they progressed into metaphase (D) and anaphase (E) by 60 min after release from nocodazole. **(F)** Quantification of 12.0 dpc PGCs in prometaphase before or after nocodazole treatment. **(G)** Quantification of 12.0 dpc PGCs entering into metaphase and anaphase after release from nocodazole at different time points. DNA was stained with DAPI. Scale bar = 10 µm. 'n' represents the total number of GFP-positive PGCs analyzed in three embryos. (**H-J**) Block at metaphase-like stage upon nocodazole plus MG132 culture. The experiments were repeated three times each, and representative images are shown.

Figure S5. *Mastl*$^{-/-}$ PGCs displayed abnormal nuclei at 12.5 dpc. (A and B) Staining for MVH (red) and GFP (green) displaying micronuclei in *Mastl*$^{-/-}$ PGCs at 12.5 dpc (B, arrows, MN). *Mastl*$^{+/+}$ PCGs had normal nuclei (A, arrows). **(C)** Quantification of abnormal nuclei in

12.5 dpc *Mastl*$^{+/+}$ and *Mastl*$^{–/–}$ PGCs. **(D)** Morphological analysis showing abnormal nuclei and giant cells in *Mastl*$^{–/–}$ PGCs at 12.5 dpc (arrowheads). PGCs were stained for GFP (green), and the DNA was stained with DAPI. Scale bar = 10 µm. 'n' represents the total number of GFP-positive PGCs analyzed in three embryos of each genotype. The experiments were repeated three times each, and representative images are shown. MN = micro-nuclei.

Figure S6. The DNA damage response pathway is activated in *Mastl*-null PGCs. (A–D) Staining for pATM S1981 (A, red, arrows) and Chk2 (C, red, arrows) in 12.5 dpc *Mastl*$^{+/+}$ PGCs. *Mastl*$^{–/–}$ PGCs displayed increased pATM S1981 (B, red, arrows) and Chk2 (D, red, arrows) staining, indicating activation of the DNA response pathway. **(E)** Quantification showing a significantly higher number of Chk2-positive *Mastl*$^{–/–}$ PGCs compared to *Mastl*$^{+/+}$ PGCs in 12.5 dpc female embryonic gonads (in %). **(F–G)** Staining for γH2AX in 12.5 dpc *Mastl*$^{+/+}$ PGCs (F, red). *Mastl*$^{–/–}$ PGCs displayed increased γH2AX staining (G, red, arrows). **(H–I)** Staining indicating increased p53-positive *Mastl*$^{–/–}$ PGCs (I, red, arrows) compared to *Mastl*$^{+/+}$ PGCs at 12.5 dpc (H, red). **(J–K)** Quantification showing significantly higher numbers of γH2AX-positive (J) and p53-positive (K) *Mastl*$^{–/–}$ PGCs compared to *Mastl*$^{+/+}$ PGCs in 12.5 dpc female gonads (in %). **(L–M)** Staining for PUMA in 12.5 dpc *Mastl*$^{+/+}$ PGCs (L, red). *Mastl*$^{–/–}$ PGCs displayed increased PUMA (M, red, arrows) expression. **(N–O)** Staining for active caspase-3 in *Mastl*$^{–/–}$ PGCs (O, red, arrows) and *Mastl*$^{+/+}$ PGCs at 12.5 dpc (N, red). **(P–Q)** Quantification showing increased numbers of PUMA-positive and active caspase-3-positive *Mastl*$^{+/+}$ and *Mastl*$^{–/–}$ PGCs in 12.5 dpc female gonads (in %). PGCs were co-stained with GFP antibody that recognizes mG (green, arrows). The DNA was stained with DAPI (blue, arrows). Scale bar = 10 µm. 'n' represents the total number of GFP-positive PGCs analyzed in three embryos of each genotype. The experiments

were repeated three times each, and representative images are shown. Data are means ± SEM, * $p < 0.05$, ** $p < 0.01$, and **** $p < 0.0001$.

Figure S7. Deletion of *Cdk1* causes loss of PGCs. (A-F) Morphological analysis of 11.5 dpc (A and B, arrowheads), 12.5dpc (C and D, arrowheads), and 13.5 dpc (E and F, arrowheads) GFP-positive PGCs in PGC-*Cdk1*$^{+/+}$ and PGC-*Cdk1*$^{-/-}$ female gonads. Scale bar = 100 µm. **(G)** Quantification of the average numbers of GFP-positive *Cdk1*$^{+/+}$ (solid line) and *Cdk1*$^{-/-}$ PGCs (dotted line) per female embryo at 11.5, 12.5, and 13.5 dpc. At 13.5 dpc, PGC-*Cdk1*$^{-/-}$ female embryonic gonads contained fewer PGCs compared to PGC-*Cdk1*$^{+/+}$ female embryonic gonads, indicating a failure to proceed through the cell cycle. The experiments were repeated three times each, for each time point gonads from one embryo of each genotype were used, and representative images are shown. Data are means ± SEM, **** $p < 0.0001$.

Figure S8. The absence of *Cdk1* in 9.5 dpc PGCs prevents mitotic entry. (A-D) Staining of the cell proliferation marker Ki67 (red, A–B, arrowheads) and the mitotic marker pHH3 S10 (red, C–D, arrowheads) in 12.5 dpc *Cdk1*$^{+/+}$ and *Cdk1*$^{-/-}$ PGCs. **(E)** Quantification of the number of *Cdk1*$^{+/+}$ and *Cdk1*$^{-/-}$ PGCs at 12.5 dpc for pHH3 S10 (in %) indicating that Cdk1 is essential for mitotic entry. **(F–G)** FACS plots depicting the percentages of *Cdk1*$^{+/+}$ and *Cdk1*$^{-/-}$ PGCs in G1 (41.2% versus 55.5%), S (20.4% versus 17.8%), and G2 (37.5% versus 24.3%) phases of cell cycle at 12.5 dpc, suggesting that the loss of Cdk1 in PGCs caused G1 arrest. The vertical axis represents the number of cells, and the horizontal axis represents the DNA content (PI staining). **(H–I)** Staining of active caspase-3 (red, arrowheads) in 12.5 dpc *Cdk1*$^{+/+}$ and *Cdk1*$^{-/-}$ PGCs, indicating upregulation of the apoptotic pathway in *Cdk1*$^{-/-}$ PGCs. **(J)** Quantification of active caspase-3-positive *Cdk1*$^{-/-}$ PGCs compared to *Cdk1*$^{+/+}$ PGCs in

12.5 dpc female embryonic gonads. PGCs were co-stained with a GFP antibody that recognizes mG (green). The DNA was stained with DAPI. Scale bars = 10 μm. ‘n’ represents the total number of GFP-positive PGCs analyzed in three embryos of each genotype. The experiments were repeated three times each, and representative images are shown. Data are means ± SEM, * $p < 0.05$.

Figure S1

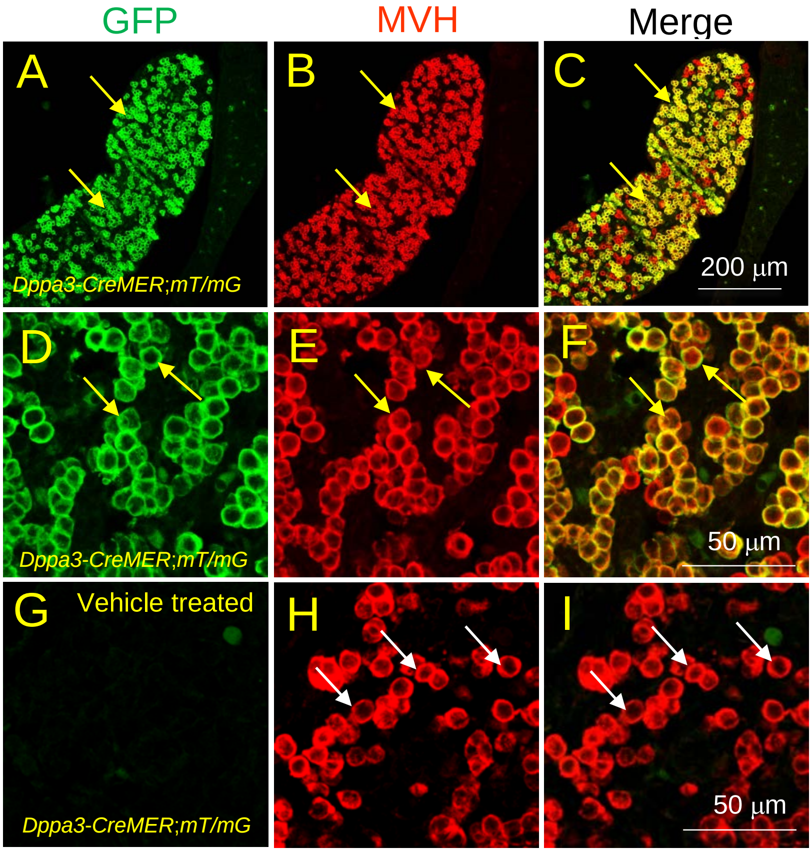

TAM (IP) 9.5 dpc →13.5 dpc

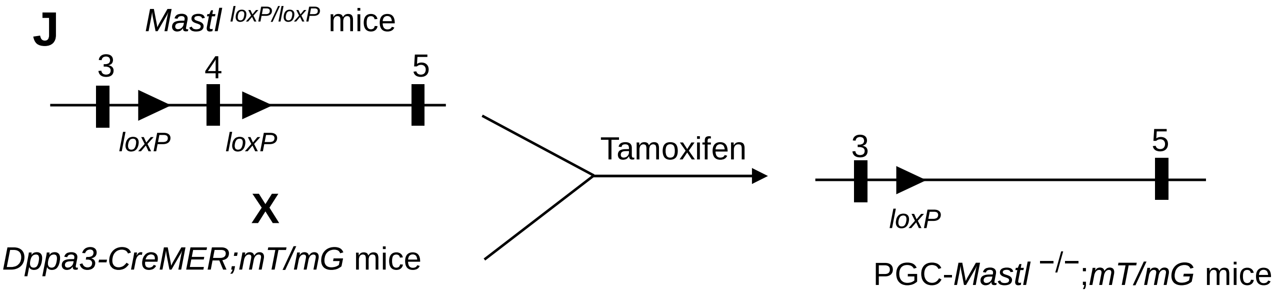

Figure S2

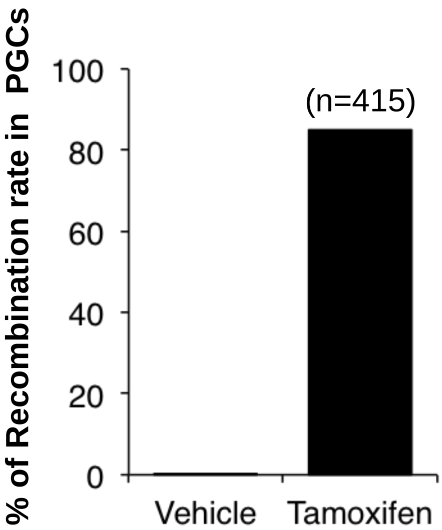

% of Recombination rate in PGCs
100
80
60
40
20
0
(n=415)
Vehicle
Tamoxifen

Figure S3

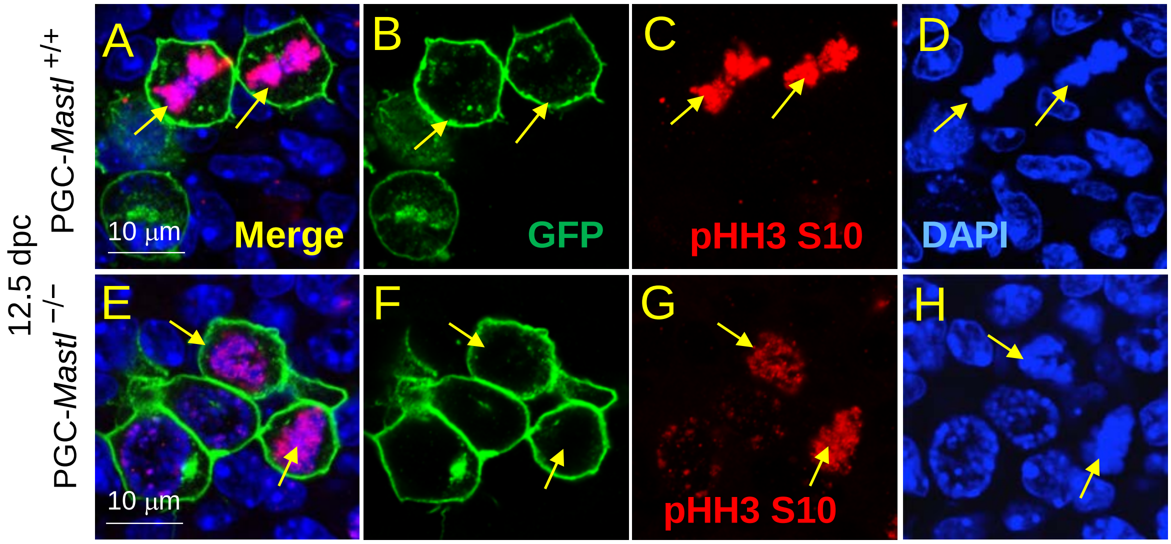
A
B
C
D
E
F
G
H
12.5 dpc
PGC-Mastl +/+
PGC-Mastl −/−
10 µm
Merge
GFP
pHH3 S10
DAPI
10 µm
pHH3 S10

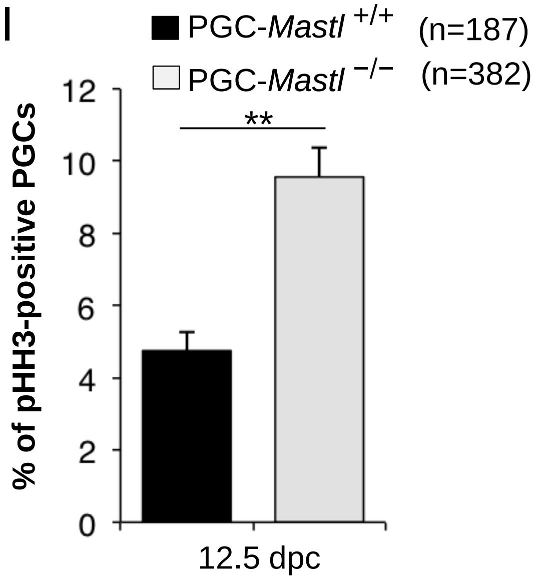
I
PGC-Mastl +/+ (n=187)
PGC-Mastl −/− (n=382)
% of pHH3-positive PGCs
12
10
8
6
4
2
0
**
12.5 dpc

Figure S4

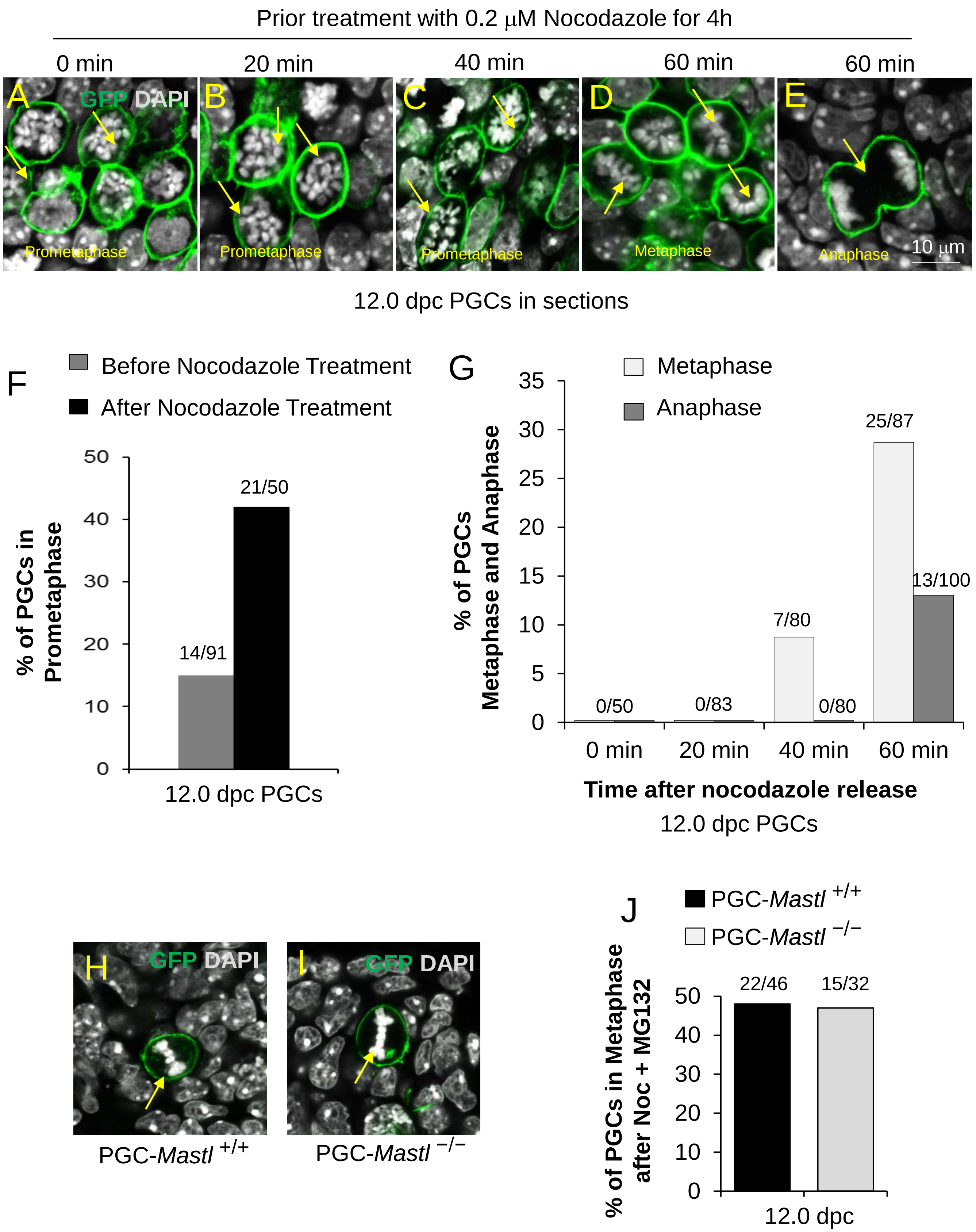

Prior treatment with 0.2 μM Nocodazole for 4h
0 min
20 min
40 min
60 min
60 min
A
GFP DAPI
Prometaphase
B
Prometaphase
C
Prometaphase
D
Metaphase
E
Anaphase
10 μm
12.0 dpc PGCs in sections
F
Before Nocodazole Treatment
After Nocodazole Treatment
% of PGCs in Prometaphase
50
40
30
20
10
0
14/91
21/50
12.0 dpc PGCs
G
Metaphase
Anaphase
% of PGCs Metaphase and Anaphase
35
30
25
20
15
10
5
0
0/50
0/83
7/80
0/80
25/87
13/100
0 min
20 min
40 min
60 min
Time after nocodazole release
12.0 dpc PGCs
H
GFP DAPI
PGC-Mastl +/+
I
GFP DAPI
PGC-Mastl −/−
J
PGC-Mastl +/+
PGC-Mastl −/−
% of PGCs in Metaphase after Noc + MG132
50
40
30
20
10
0
22/46
15/32
12.0 dpc

Figure S5

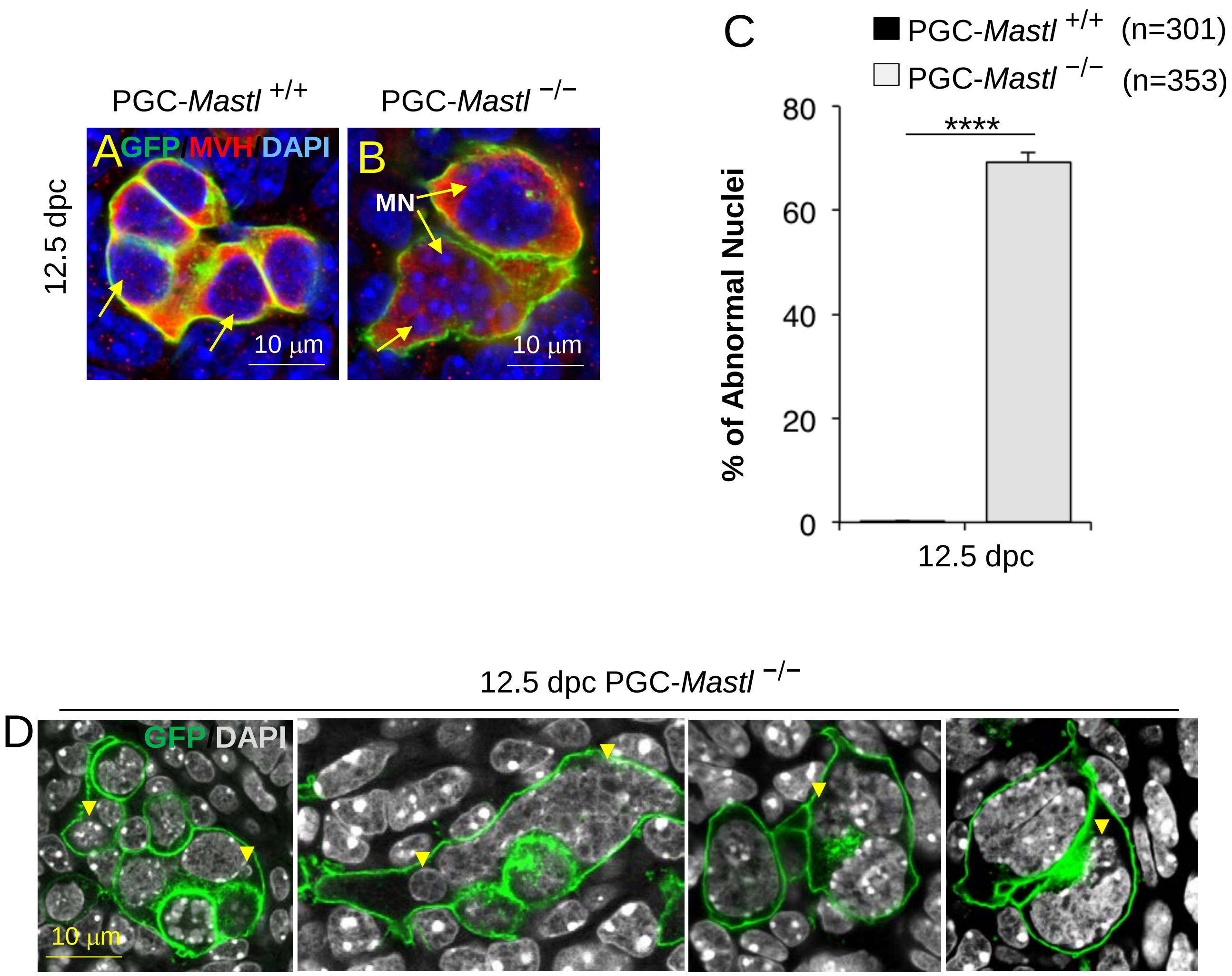

PGC-*Mastl* +/+
PGC-*Mastl* −/−
12.5 dpc
A GFP/MVH/DAPI
B
MN
10 µm
10 µm
C
PGC-*Mastl* +/+ (n=301)
PGC-*Mastl* −/− (n=353)
% of Abnormal Nuclei
80
60
40
20
0
****
12.5 dpc
12.5 dpc PGC-*Mastl* −/−
D
GFP/DAPI
10 µm

Figure S6

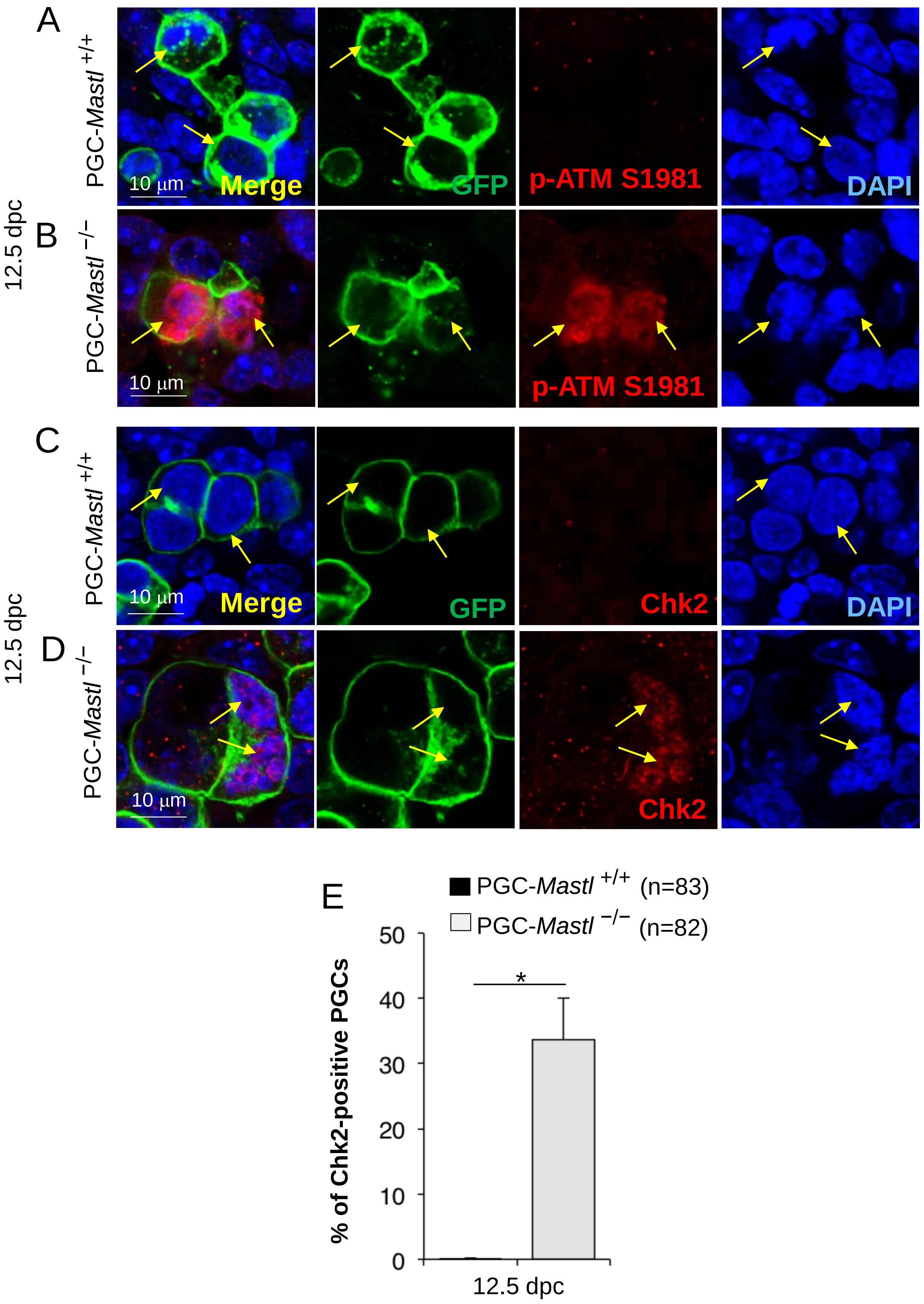
A
B
C
D
E
12.5 dpc
PGC-Mastl +/+
PGC-Mastl −/−
10 μm
Merge
GFP
p-ATM S1981
DAPI
Chk2
PGC-Mastl +/+ (n=83)
PGC-Mastl −/− (n=82)
% of Chk2-positive PGCs
50
40
30
20
10
0
*
12.5 dpc

Figure S6 contd….

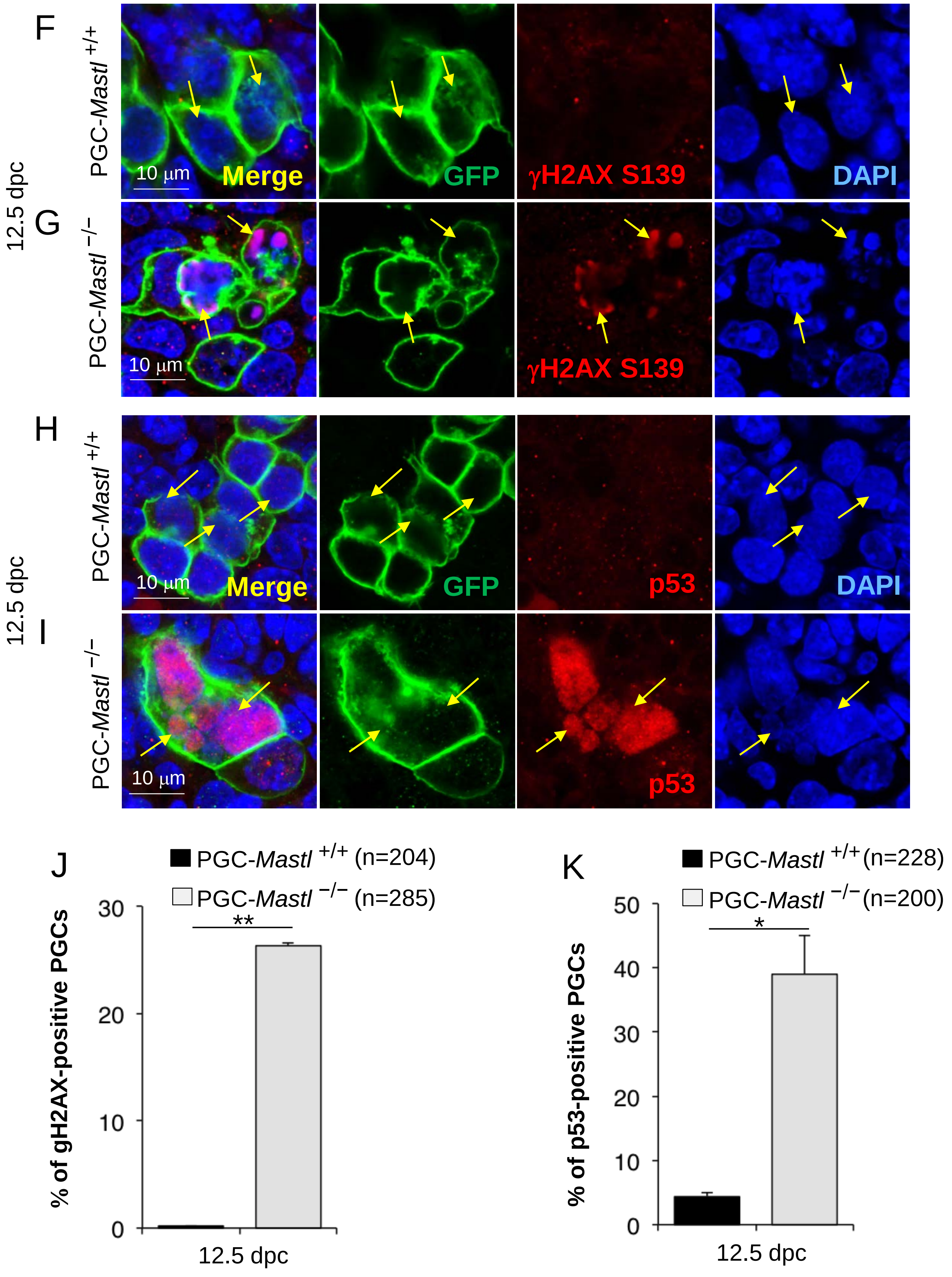

Figure S6 contd….

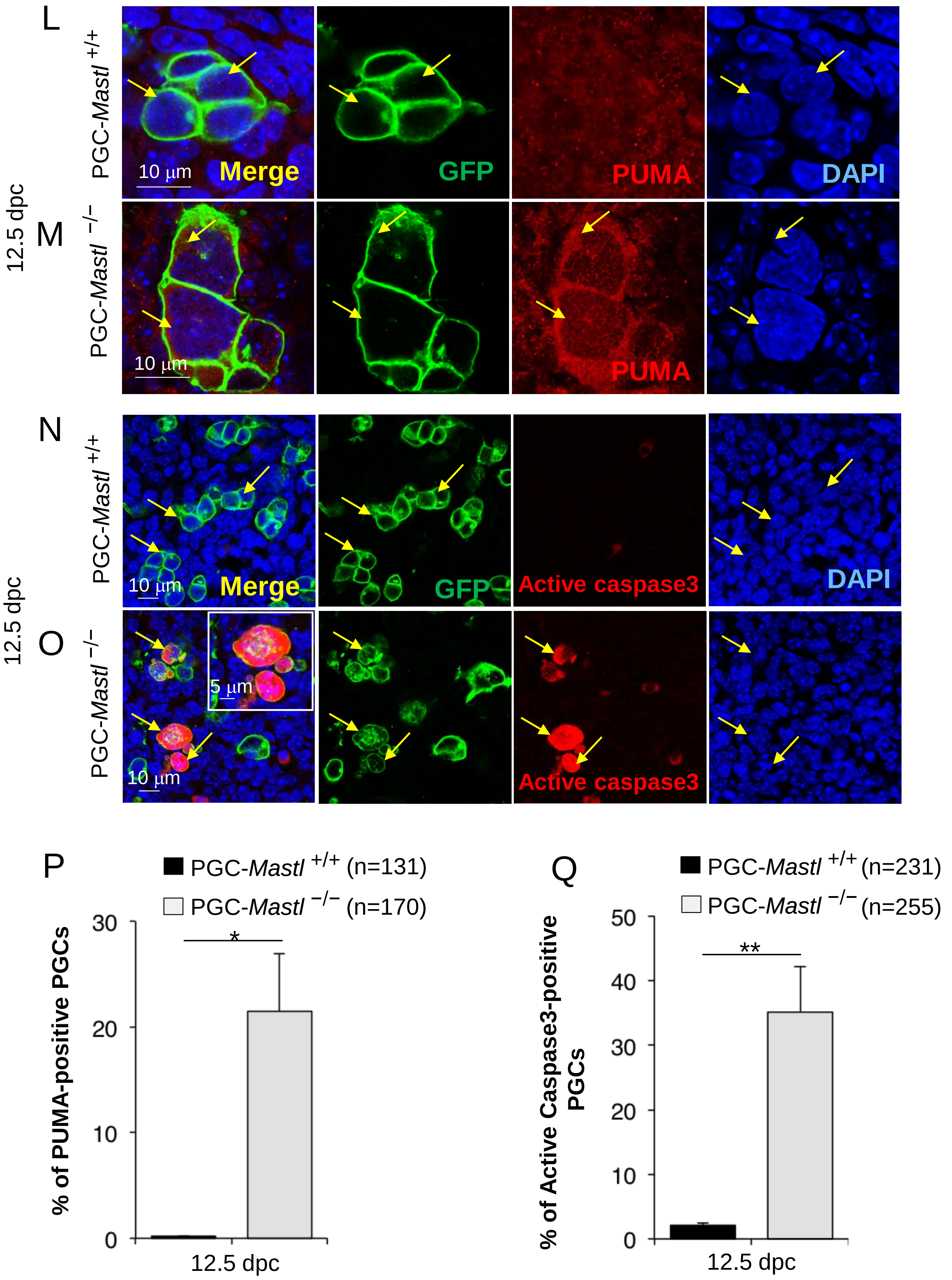

Figure S7

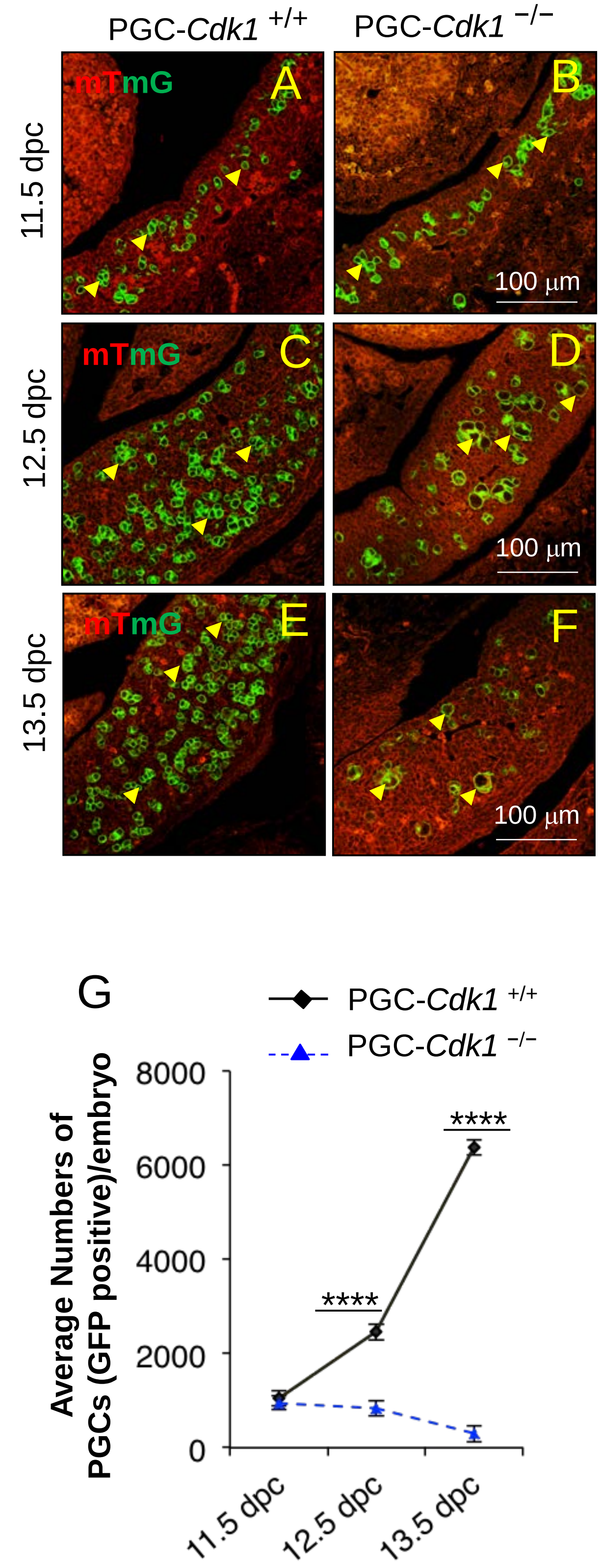

PGC-Cdk1 +/+
PGC-Cdk1 −/−
11.5 dpc
12.5 dpc
13.5 dpc
mTmG
A
B
C
D
E
F
100 µm
100 µm
100 µm
G
PGC-Cdk1 +/+
PGC-Cdk1 −/−
Average Numbers of PGCs (GFP positive)/embryo
8000
6000
4000
2000
0
****
****
11.5 dpc
12.5 dpc
13.5 dpc

Figure S8

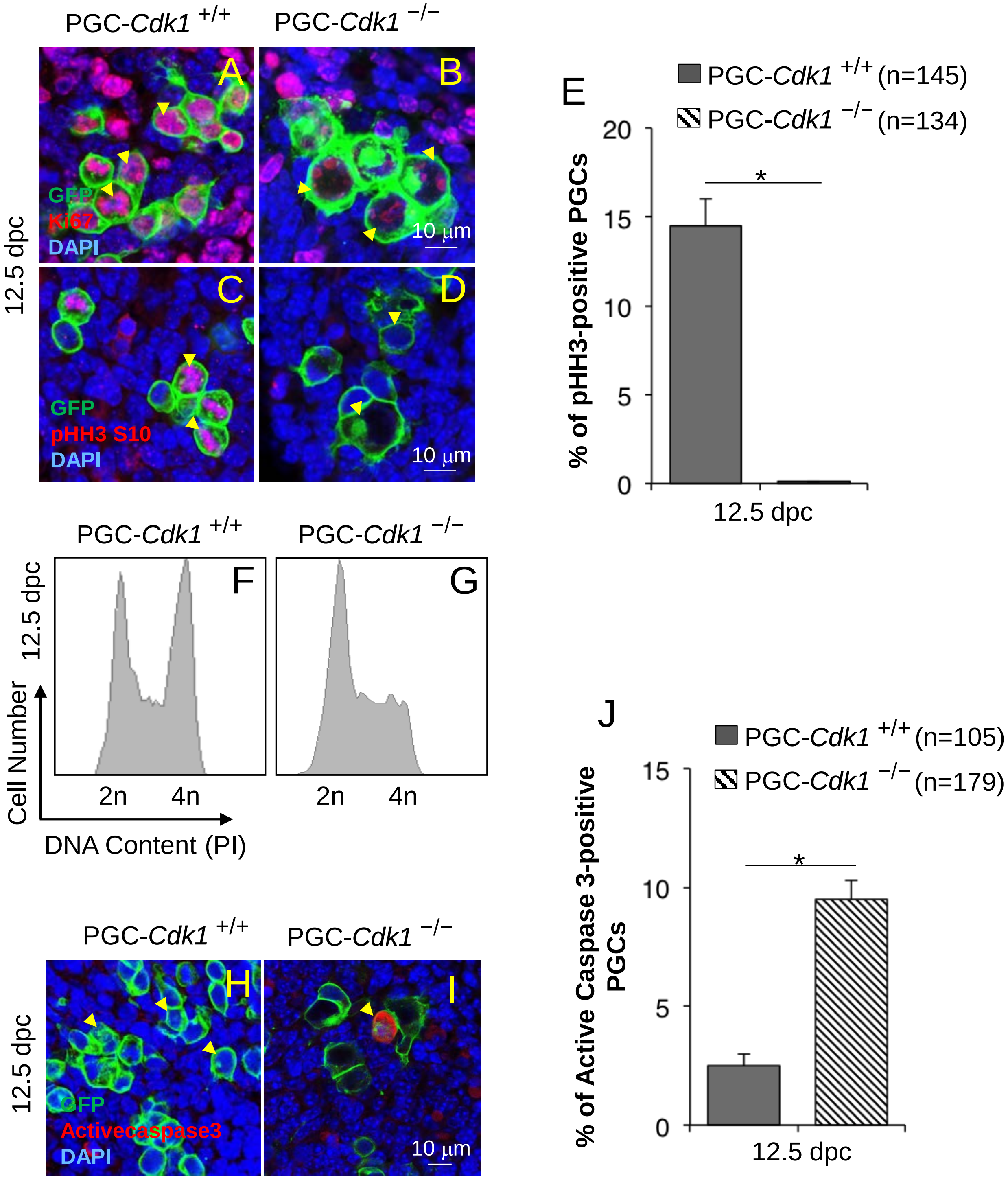
PGC-Cdk1 +/+
PGC-Cdk1 −/−
A
B
C
D
GFP
Ki67
DAPI
12.5 dpc
10 µm
GFP
pHH3 S10
DAPI
10 µm
E
PGC-Cdk1 +/+ (n=145)
PGC-Cdk1 −/− (n=134)
% of pHH3-positive PGCs
20
15
10
5
0
*
12.5 dpc
PGC-Cdk1 +/+
PGC-Cdk1 −/−
F
G
12.5 dpc
Cell Number
2n
4n
2n
4n
DNA Content (PI)
PGC-Cdk1 +/+
PGC-Cdk1 −/−
H
I
12.5 dpc
GFP
Activecaspase3
DAPI
10 µm
J
PGC-Cdk1 +/+ (n=105)
PGC-Cdk1 −/− (n=179)
% of Active Caspase 3-positive PGCs
15
10
5
0
*
12.5 dpc
